# Research Hotspots and Effectiveness of Transcranial Magnetic Stimulation in Pain: A Bibliometric Analysis

**DOI:** 10.3389/fnhum.2022.887246

**Published:** 2022-06-21

**Authors:** Chong Li, Mingyu Sun, Shiliu Tian

**Affiliations:** ^1^School of Kinesiology, Shanghai University of Sport, Shanghai, China; ^2^Key Laboratory of Exercise and Health Science of Ministry of Education, Shanghai University of Sport, Shanghai, China; ^3^Shanghai Frontiers Science Research Base of Exercise and Metabolic Health, Shanghai, China; ^4^Fujian Sports Vocational Education and Technical College, Fuzhou, China

**Keywords:** transcranial magnetic stimulation, pain, citation burst, Web of Science, CiteSpace

## Abstract

Transcranial magnetic stimulation, as a relatively new type of treatment, is a safe and non-invasive method for pain therapy. Here, we used CiteSpace software to visually analyze 440 studies concerning transcranial magnetic stimulation in pain research from 2010 to 2021, indexed by Web of Science, to clarify the research hotspots in different periods and characterize the process of discovery in this field. The United States ranked first in this field. Lefaucheur JP, Fregni F, and Andrade ACD made great contributions to this field of study. The most prolific institution was University of São Paulo. The four main hot keywords were neuropathic pain, motor cortex, connectivity, and non-invasive brain stimulation. There were three main points that were generally accepted: (1) definite analgesic effect of high-frequency rTMS of M1 contralateral to pain side in neuropathic pain; (2) there are inconclusive recommendations regarding rTMS of the dorsolateral prefrontal cortex (DLPFC) in fibromyalgia and neuropathic pain; (3) there is low-quality evidence that single doses of high-frequency rTMS of the motor cortex may have short-term effects on chronic pain. This bibliometric analysis indicated that prospective, multi-center, large-sample, randomized controlled trials are still needed to further verify the effectiveness of various transcranial magnetic stimulation parameters in pain research.

## Introduction

Pain is termed as an unpleasant sensory and emotional experience associated with, or resembling that associated with, actual or potential tissue damage (Raja et al., 2020). Pain is a subjective emotional experience, and there are few effective treatments. At present, application of analgesic drugs is the main way to relieve pain (Klit et al., 2009; Alles and Smith, 2018). However, long-term use of analgesic drugs is not only prone to addiction, but also has many side effects (Koob, 2021). Transcranial magnetic stimulation (TMS) is considered to be a safe and non-invasive treatment method that has been extensively used in pain therapy (Leung et al., 2009; de Andrade et al., 2011; O’Connell et al., 2014). Different frequencies of TMS can achieve different therapeutic purposes.

Studies of the motor cortex indicate that high frequencies (>1 Hz) mainly produce excitatory effects, while low-frequency stimulation (≤ 1Hz) produces inhibitory effects (Hallett, 2007; Pitcher et al., 2021). TMS can affect local nerves by altering neural function at multiple sites through the connectivity and interactions between neural networks (Nurmikko et al., 2016; Li et al., 2021). Thus, TMS may have therapeutic effects on pain intensity resulted from various diseases.

Visualization analysis is to use of relevant visualization software to import and convert a large amount of literature data into a visual atlas, so that readers can have a more intuitive and clear understanding of the data contained in the literature through the atlas (Chen, 2004). Based on co-citation analysis theory and pathfinding network algorithm, CiteSpace software can analyze literature of specific disciplines or fields from multiple perspectives and draw visual maps, so as to explore the critical paths, research hotspots, and frontiers of the evolution of this discipline or field (Chen and Song, 2019). In recent years, using CiteSpace software combined with relevant authoritative databases to analyze the literature visualization of a certain discipline or field has become a hot research topic for scholars all over the world (Chen et al., 2012; Ugolini et al., 2013; Xu and Sun, 2020; Wang et al., 2021).

The aim of this study was two-folded: (1) perform a visual analysis of TMS in pain studies using CiteSpace software, and (2) objectively clarify the time changes of research hotspots and dynamic frontiers in this field.

## Materials and Methods

### Data Source and Search Strategy

Published papers were retrieved via a topic search of Web of Science (WOS) Core Collection Database. The search terms were as follows: (((((TS = (transcranial magnetic stimulation)) OR TS = (TMS)) OR TS = (rTMS)) OR TS = (iTBS)) OR TS = (cTBS)) AND TS = (pain). Time span were retrieved from January 01, 2010 to December 31, 2021.

### Inclusion Criteria

**FIGURE 1:**
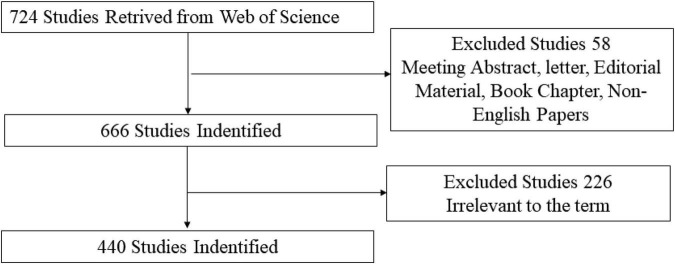
Flow chart of studies inclusion.

Studies related to the application of TMS in pain research were selected after reading the title and abstract. Only articles and reviews were included. Other document types, such as letters, commentaries, and meeting abstracts, were excluded. In addition, the publication language was restricted to English. The flow chart of the inclusion is shown in Figure 1. Finally, 440 records (344 articles, 96 reviews) were used in the final analysis.

### Analytic Methods

#### Software Parameter Settings

CiteSpace is a bibliometric analysis visualization software developed by Prof. Chen Chaomei (Drexel University, United States) for bibliometric analysis. We used CiteSpace 5.8.R3 to analyze the final records. The “Time Sliding” value was set to 1 year and the type of Node was selected according to the purpose of analysis.

#### Interpretation of Main Parameters in Visualization Map

##### Citation Tree Rings

The citation tree ring represents the citation history of a paper. The color of a citation ring denotes the time of the corresponding citation, and the thickness of an annual ring is directly proportional to the number of citations in the corresponding time sliding.

##### Node Circle and the Link Between Nodes

The radius of a node circle indicates the number of papers published in the author or institutional co-authorship network, and also indicates the frequency of keywords in the co-occurrence network. A link indicates the presence of co-authorship or a co-occurrence relationship. The node colors range from cold to warm to represent the chance of time, blue for earlier years, and red for recent years.

##### Betweenness Centrality

Betweenness centrality is an index that measures the importance of nodes in the network. CiteSpace uses this index to discover and measure the importance of studies and highlights such studies with purple circles.

##### Cluster View and Burst Detection

Cluster view is carried out on the generated map, and each cluster is labeled by citing the title, keywords, and subject headings in the abstract of the citing reference. The function of Burst detection is to detect the situation where there is a great change in the number of citations in a certain period. Thus it can be used to find the decline or rise of keywords.

##### Dual-Map Overlaps

Dual-map overlaps are a new method to display the distribution and citation trajectory of papers in various disciplines. As a result, there is a distribution of citing journals on the left side and a distribution of cited journals on the right side. The curve is the citation line, which completely shows the context of the citation.

## Results

### Publication Outputs

**FIGURE 2:**
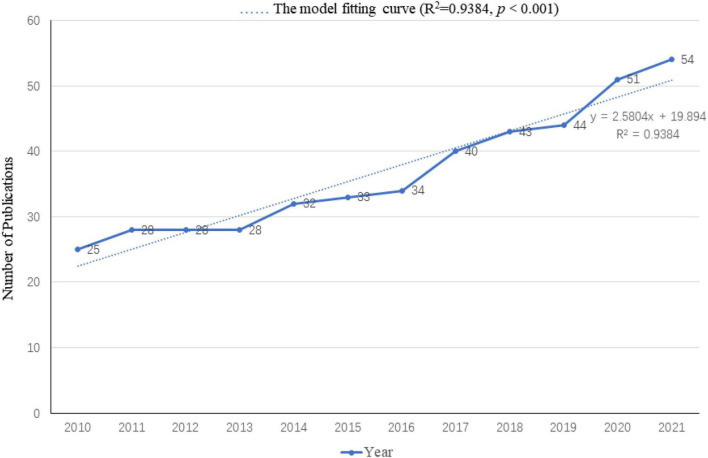
Annual publication outputs and the model fitting curve of the time trend of transcranial magnetic stimulation (TMS) in pain research.

A total of 440 publications were included in the analysis. Figure 2 shows the distribution of the annual publication of TMS in pain research from 2010 to 2021. The overall trend is positive and the time trend of publications indicated a significant correlation (R^2^ = 0.9384, *p* < 0.001) between the annual publication outputs and the years in the last 11 years.

### Journal Co-citation Analysis

**FIGURE 3:**
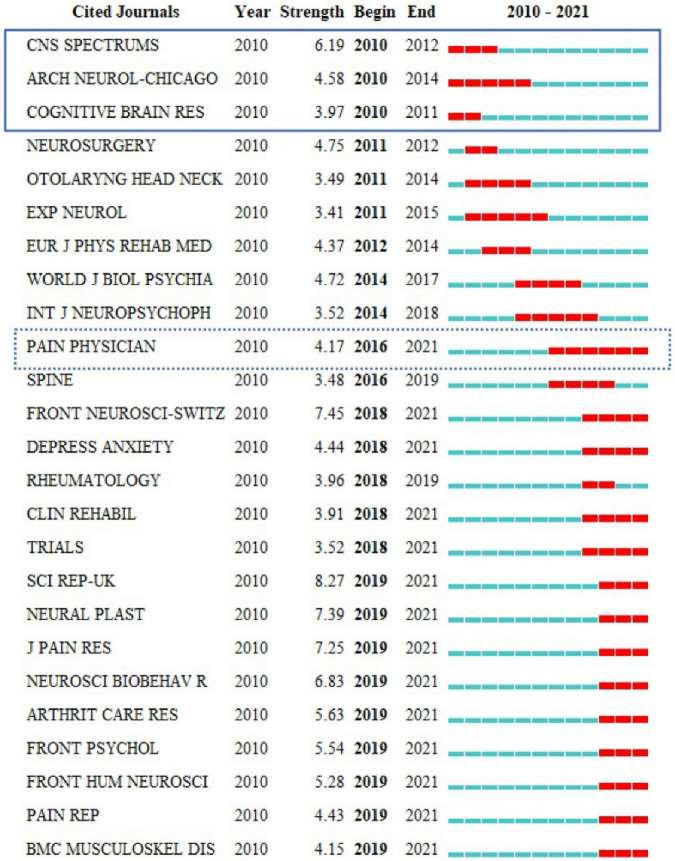
Top 25 cited journals with the strongest citation burst.

**FIGURE 4:**
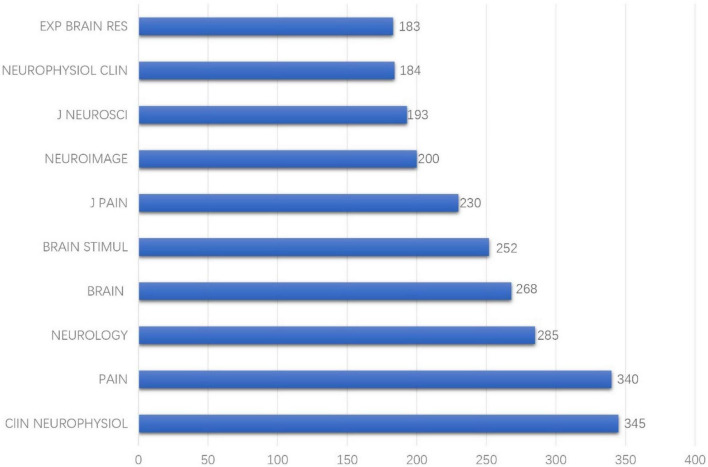
Top 10 most cited journals among 440 studies published from 2010 to 2021.

Journal co-citation analyses of reference from 2010 to 2021 cited by 440 publications found that among the earliest journals, *CNS SPECTRUMS*, *ARCH NEUROL-CHICAGO*, and *COGNITIVE BRAIN RES* had the earliest hotspots in 2010, and *PAIN PHYSICIAN* had hotspots for the longest period and also had recent frontier hotspot from 2016 to 2021 (Figure 3). Among the top 10 cited journals, *CLIN NEUROPHYSIOL* was the most frequently cited, which was cited 345 times, followed by *PAIN* (340 times) and *NEUROLOGY* (285 times) (Figure 4).

**FIGURE 5:**
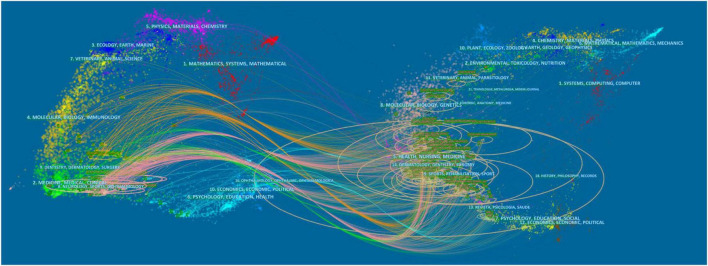
Visualization of dual-map overlays of citing journals and cited journals of 440 studies published from 2010 to 2021. The colored curve indicates the path of citation, which originates from 11 fields of the citing journals on the left and points to 14 fields of the cited journals on the right.

Based on the Blondel algorithm, dual-map overlaps of journals are displayed in Figure 5. The citing journals of 440 studies were mainly from the fields of MEDICINE, MEDICAL, NEUROLOGY, and SPORTS. The cited journals were mainly from the fields of HEALTH, MEDICINE, SPORTS, and REHABILITATION. As shown in the center of the circle on the right, rehabilitation medicine was the most concentrated one in the cited journals. While in the center of the circle on the left, neurology medicine was the hotspot of current research on TMS in pain research.

### Reference Co-citation Analysis

**FIGURE 6:**
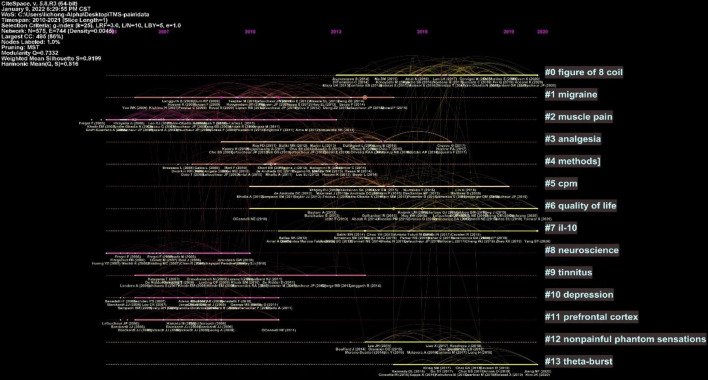
Timeline view of reference co-citation analysis.

The clustered research categories of reference co-citation analysis were divided into 14 groups (#0-13). The timeline view of clusters was shown in Figure 6, which presents the characteristics of the time-span citation information for the cluster domains. The cluster category with the largest time span for the cited references was #1 migraine from 2006 to 2015, which was also the most frequently cited category. Moreover, there were a series of important landmark achievements in this cluster. Rossi et al. (2009) released guidelines for the use of TMS in clinical practice and research. Lipton and Pearlman (2010) published a review of TMS in the treatment of migraine. Lefaucheur et al. (2011) assessed the value of rTMS in the prediction of the efficacy of epidural motor cortex stimulation to treat neuropathic pain. Lefaucheur et al. (2014) released evidence-based guidelines on the therapeutic use of rTMS.

The top 10 cited reference information of the 440 included studies are summarized in Table 1. The studies by Lefaucheur et al. (2014); Klein et al. (2015), Rossini et al. (2015) were guidelines for the efficacy and safety of TMS in clinical research. A study by Mhalla et al. (2011) focused on the long-term maintenance of the analgesic effects of TMS in fibromyalgia. A study by Hosomi et al. (2013) was a randomized crossover sham-controlled trial focusing on the effect of daily rTMS of primary motor cortex for neuropathic pain. A study by de Andrade et al. (2011) investigated the role of endogenous opioid systems in the analgesic effects induced by rTMS. A study by Leung et al. (2009) was a meta-analysis of rTMS for suppressing neuropathic pain.

**TABLE 1:** Ten representative studies of transcranial magnetic stimulation (TMS) in pain research among the cited references of the included 440 studies.

Study	Citation counts	Journal	Study type	Sample	Intervention	Outcomes	Highlights
Lefaucheur et al., 2014	67	Clin Neurophysiol	Guidelines	\	\	\	Recommendation: definite analgesic effect of HF rTMS of M1 contralateral to pain side in neuropathic pain (Level A)
Mhalla et al., 2011	38	Pain	Randomized controlled trial	40	40 fibromyalgia patients were randomized to receive active or sham rTMS of the left primary motor cortex.	Self-reported average pain intensity with the numerical scale.	TMS may be a valuable and safe new therapeutic option in patients with fibromyalgia.
Rossini et al., 2015	35	Clin Neurophysiol	An updated report	\	\	\	Further research is still needed to compare the respective value of various cortical targets, depending on the side and frequency of stimulation and the clinical presentation, with respect to the location and the respective sensory-discriminant and affective-emotional components of pain.
Hosomi et al., 2013	31	Pain	A randomized, multicenter, double-blind, crossover, sham-controlled trial.	70	A series of 10 daily 5-Hz rTMS (500 pulses/session) of primary motor cortex (M1) or sham stimulation was applied to each patient with a follow-up of 17 days.	McGill pain questionnaire.	Daily high-frequency rTMS of M1 is tolerable and transiently provides modest pain relief in neuropathic pain patients.
de Andrade et al., 2011	29	Pain	A randomized, double-blind crossover design.	12	Three groups of 12 volunteers were selected at random and given active stimulation (frequency 10Hz, at 80% motor threshold intensity, 1500 pulses per session) of the right M1, active stimulation of the right DLPFC, or sham stimulation, during two experimental sessions 2 weeks apart.	Cold pain thresholds and the intensity of pain.	Endogenous opioids are shown to be involved in the analgesic effects of repetitive transcranial magnetic stimulation of the motor cortex.
Klein et al., 2015	28	Pain	Guidelines	\	\	\	The suffering and disability associated with uncontrolled chronic pain, the common and serious adverse effects associated with pain medications, and the preliminary evidence of efficacy and safety of TMS for treating some types of pain mandate greater investment in developing this therapy.
Rossi et al., 2009	26	Clin Neurophysiol	Guidelines	\	\	\	The present updated guidelines review issues of risk and safety of TMS in clinical practice and research.
Moisset et al., 2016	26	European Journal of Pain	Review	\	\	\	LTP-like mechanisms, dependence on endogenous opioids and increase in concentration of neurotransmitters (monoamines, GABA) have all been implicated in its analgesic effects.
Leung et al., 2009	26	The Journal of Pain	A meta-analysis	\	\	\	rTMS appears to be more effective in suppressing centrally than peripherally originated neuropathic pain states.
O’Connell et al., 2014	25	The Cochrane database of systematic reviews	An updated review	\	\	\	The available evidence suggests that low-frequency rTMS, rTMS applied to the pre-frontal cortex, CES and tDCS are not effective in the treatment of chronic pain.

### Innovative Reference Analysis

The Sigma value can be used to identify innovative references. Five innovative references are summarized in Table 2. A study by Lefaucheur et al. (2011) was a retrospective study that assess the value of rTMS to predict the efficacy of epidural motor cortex stimulation to treat neuropathic pain. A study by de Oliveira et al. (2014) found that rTMS of the premotor cortex/dorsolateral prefrontal cortex was not effective in relieving central poststroke pain. A study by Lindholm et al. (2015) found that the right S2 cortex is a promising new target for the treatment of neuropathic orofacial pain with high-frequency rTMS. Kang et al. (2009) found that the therapeutic efficacy of rTMS was not demonstrated when rTMS was applied to the hand motor cortical area in patients with chronic neuropathic pain at multiple sites in the body. A study by Picarelli et al. (2010) was a controlled randomized trial that highlighted an add-on therapy of high-frequency rTMS for refractory CRPS type I patients.

**TABLE 2:** Five innovative studies about TMS in pain research among the cited references of the included 440 studies.

Study	Sigma*	Journal	Study type	Sample	Intervention	Outcomes	Highlights
Lefaucheur et al., 2011	0.14	Journal of Pain	Retrospective study	59	Patients were treated by epidural motor cortex stimulation for more than 1 year and in whom active and sham 10 Hz rTMS sessions were performed targeted over the cortical representation of the painful area.	The visual analog scale	Neuropathic pain can be significantly relieved by motor cortex rTMS.
de Oliveira et al., 2014	0.13	Journal of Pain	Prospective, double-blind, placebo-controlled study	23	Active rTMS and sham rTMS, and were treated with 10 daily sessions of rTMS over the left PMC/DLPFC (10 Hz, 1,250 pulses/d).	The visual analog scale	rTMS of the PMC/DLPFC is not effective in relieving CPSP.
Lindholm et al., 2015	0.13	Pain	Randomized, placebo-controlled, crossover study	16	Navigated high-frequency rTMS was given to the sensorimotor (S1/M1) and the right secondary somatosensory (S2) cortices.	The numerical rating scale	The right S2 cortex is a promising new target for the treatment of neuropathic orofacial pain with high-frequency rTMS.
Kang et al., 2009	0.13	Archives of Physical Medicine and Rehabilitation	Blinded, randomized crossover study	11	rTMS was applied on the hand motor cortical area using a figure-of-eight coil. One thousand stimuli were applied daily on 5 consecutive days. Real and sham rTMS were separated by 12 weeks.	Numeric rating scale, the Brief Pain Inventory	The therapeutic efficacy of rTMS was not demonstrated when rTMS was applied to the hand motor cortical area in patients with chronic neuropathic pain at multiple sites in the body, including the lower limbs, trunk, and pelvis.
Picarelli et al., 2010	0.11	Journal of Pain	Double-blind, placebo-controlled, randomized trial	23	Patients were treated with the best medical treatment (analgesics and adjuvant medications, physical therapy) plus 10 daily sessions of either real or sham 10 Hz rTMS to the motor cortex (M1).	The visual analog scale	Repetitive sessions of high-frequency rTMS shows efficacy as an add-on therapy to refractory CRPS type I patients.

*Sigma = (centrality+1)burstness (burstness on the index) to identify innovative reference.

### Analysis of Keywords

**FIGURE 7:**
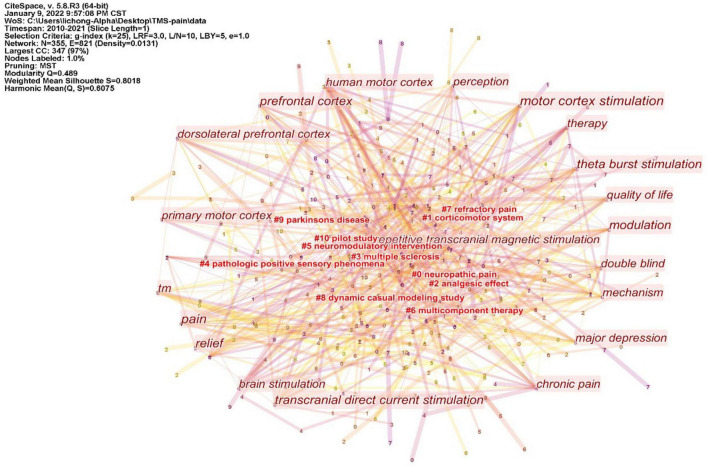
Cluster of keywords from 440 inclusion studies. The keyword clusters (LLR algorithm) were divided into 11 categories (#0-10). Those without # are high-frequency keywords.

The keywords co-occurrence analysis in the 440 included studies revealed 355 keyword nodes and 821 connection lines. The keyword clusters were divided into 11 categories (#0-10) (Figure 7). The largest cluster (#0) has 53 members and a silhouette value of 0.847. It is labeled as *neuropathic pain* by LLR. The most relevant citer to the cluster is “Motor cortex stimulation for deafferentation pain” (Hussein et al., 2018). The second-largest cluster (#1) labeled as *corticomotor system* has 49 members and a silhouette value of 0.752. The most relevant citer to the cluster is “Paired associative electroacupuncture and transcranial magnetic stimulation in humans” (Huang et al., 2019). The third-largest cluster (#2) labeled as *analgesic effect* has 43 members and a silhouette value of 0.763. The most relevant citer is “Neural correlates of the antinociceptive effects of repetitive transcranial magnetic stimulation on central pain after stroke” (Ohn et al., 2012).

The ten representative keywords of TMS in pain research from 440 included studies are shown in Table 3. Studies of TMS in pain have focused on stimulating the motor cortex and dorsolateral prefrontal cortex. Existing studies have focused on pain including neuropathic pain, chronic pain, intractable deafferentation pain, and pain related to spinal cord injury. At present, more attention is paid to theta-burst stimulation.

**TABLE 3:** Ten representative keywords of TMS in pain research from 440 included studies.

Rank	Keyword	Year	Count	Centrality
1	Neuropathic pain	2010	110	0.06
2	Motor cortex	2010	102	0.05
3	Brain	2010	56	0.03
4	Modulation	2012	42	0.12
5	Theta-burst stimulation	2010	39	0.14
6	Excitability	2011	38	0.04
7	Chronic pain	2010	35	0.09
8	Dorsolateral prefrontal cortex	2010	34	0.09
9	Intractable deafferentation pain	2010	29	0.04
10	Spinal cord injury	2013	29	0.04

**FIGURE 8:**
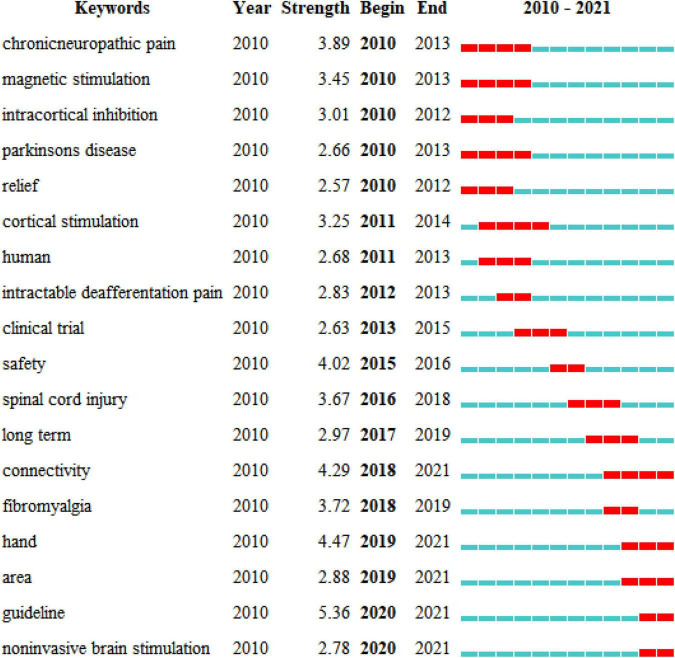
Top 18 keywords with the strongest citation bursts of the 440 included studies from 2010 to 2021.

Figure 8 shows the years when hot keywords began to appear and end. The hot keywords indicated three main points. (1) In the first stage, chronic neuropathic pain (2010−2013) was the first hot keyword. (2) In the second stage, intractable deafferentation pain (2012−2013), spinal cord injury (2016−2018), and fibromyalgia (2018−2019) were the keywords, mainly describing the effects of TMS in different pain types. (3) In the third stage, connectivity (2018−2021) and area (2019−2021) were the keywords, indicating that studies are increasingly focusing on brain mechanisms in the area of TMS in pain.

### Authoritative Authors Analysis

Authoritative authors analysis is presented in Table 4. In terms of publications number, Jeanpascal Lefaucheur and Felipe Fregni both published 19 papers separately, followed by author Daniel Ciampi De Andrade (11 publications) and Youichi Asitoh (9 publications). In terms of co-citation counts, Lefaucheur (284 citations) ranked first as the most co-cited author, followed by author Khedr EM (136 citations), Rossi S (126 citations).

**TABLE 4:** The top 10 authors and co-cited authors in TMS research in pain.

Rank	Author	Count	Co-cited author	Count
1	Jeanpascal lefaucheur	19	Lefaucheur	284
2	Felipe fregni	19	Khedr em	136
3	Daniel ciampi de andrade	11	Rossi s	126
4	Youichi asitoh	9	Andre-obadia n	122
5	Jeffrey j borckardt	8	Fregni f	100
6	Alvaro pascualleone	8	Oconnell ne	99
7	Mark s george	7	Garcia-larrea l	96
8	Alaa mhalla	7	Rossini pm	96
9	Albert leung	7	Borckardt jj	96
10	Wolnei caumo	7	Mhalla a	93

### Co-country and Co-institution Analysis

**FIGURE 9:**
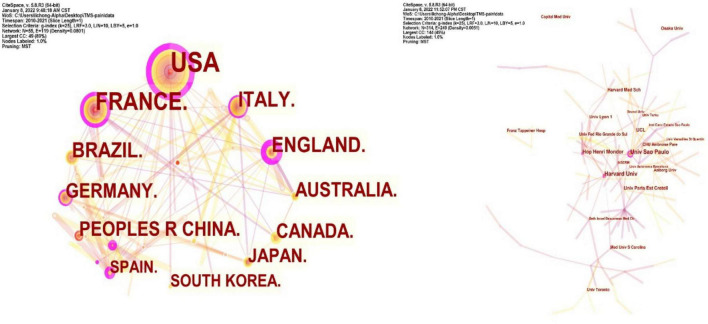
Network map of countries and institutions in TMS in pain research.

Collaboration networks of authoritative countries and institutions were presented in Figure 9. Amongst the 440 publications included in this study, the top-ranked country by citation counts was the United States (111 publications). The second one was France with citation counts of 67 and the third was Italy with citation counts of 40. In terms of authoritative institutions, Univ São Paulo (22) ranked first in the number of publications, followed by Harvard Univ (19) and Hop Henri Mondor (14), as presented in Table 5.

**TABLE 5:** The top 10 countries or institution among 440 studies.

Rank	Country	Count	Centrality	Bursts
1	United states	111	0.49	2.68
2	France	67	0.29	2.89
3	Italy	40	0.13	\
4	Brazil	37	0.06	\
5	England	37	0.40	\
6	Australia	33	0.13	\
7	Canada	33	0.06	\
8	Peoples r china	29	0.07	4.47
9	Japan	28	0.00	\
10	Spain	24	0.02	\
	**Institution**			
1	Univ sao paulo	22	0.23	\
2	Harvard univ	19	0.18	2.99
3	Hop henri mondor	14	0.11	\
4	Ucl	12	0.09	\
5	Univ pris est creteil	10	0.09	\
6	Harvard med sch	9	0.04	\
7	Univ lyon 1	9	0.03	\
8	Osaka univ	9	0.00	\
9	Univ toronto	8	0.02	\
10	Med univ s carolina	8	0.07	3.69

## Discussion

### General Trends of Transcranial Magnetic Stimulation in Pain Research

From 2010 to 2021, TMS has received great attention, and research related to pain has been increasingly performed. It is reasonable to expect a promising future for TMS in pain research based on analyzing the time trend of annual publication outputs.

Among the 10 top-performing journals, *Brain* (IF, 2021 = 13.501) had IF score > 10, and six journals, namely, *Pain* (IF, 2021 = 6.961), *Neurology* (IF, 2021 = 9.91), *Brain Stimulation* (IF, 2021 = 8.955), *Journal of Pain* (IF, 2021 = 5.828), *Neuroimage* (IF, 2021 = 6.556), *Journal of Neuroscience* (IF, 2021 = 6.167) had IF scores between 5, 000 and 10, 000. Amongst the top 10 countries, eight are developed countries and only Brazil and China are developing countries. From this perspective, there was still a wide gap between developed and developing countries in this field. The United States ranked first in terms of publication count (111) and is the leading country in terms of the over influence in this area. Among the 10 top institutions, University of São Paulo ranked first in terms of publication count (22) but it lacks international cooperation. Amongst authoritative authors, Jeanpascal Lefaucheur and Felipe Fregni both ranked first in terms of publication count (19). Jeanpascal Lefaucheur is a doctor in Henri Mondor Hospital from France and Felipe Fregni is a researcher in Harvard Medical School from the United States.

### Emerging Trends of Transcranial Magnetic Stimulation in Pain Research

The evolution of a knowledge domain can be reflected by keywords. Therefore, keywords analysis can reveal emerging trends and provide directions for future research.

(I) Neuropathic pain: Neuropathic pain refers to pain initiated or caused by a primary lesion or dysfunction in the somatosensory system (Finnerup et al., 2021). Neuropathic pain is thought to be associated with peripheral nerve problems such as diabetes, but injuries to the brain or spinal cord can also lead to chronic neuropathic pain (Cohen and Mao, 2014). As a non-invasive brain stimulation, TMS now has become a treatment for neuropathic pain. However, it is difficult to determine which specific parameters are best for clinical use. The effectiveness of TMS depends on the type of neuropathic pain, and significant results have been reported when employing rTMS at 20 Hz (Aamir et al., 2020; Attia et al., 2021). Therefore, multi-centers, large sample sizes, randomized controlled trials are needed to carry out.

(II) Motor Cortex: The most commonly targeted area of TMS in pain research is represented by the M1 contralateral to the position corresponding to the somatotopic location of the pain source (O’Connell et al., 2018). With further research, the secondary somatosensory cortex (S2) and supplementary motor area (SMA) show as promising targeted areas for pain research (Lockwood et al., 2013; Rao et al., 2020).
(III) Connectivity: The pain caused by central nervous system injury may be caused by the lack of connectivity between various parts of the brain caused by neuron damage. Regardless of the etiology and pain model, chronic pain may trigger various forms of maladaptive structural connection. TMS can strengthen the plasticity of neuronal connections. Locally, within one hemisphere, increased EEG activity can be seen in several neighboring electrodes, suggesting the spread of TMS-evoked activity to anatomically interconnected cortical areas (Martin et al., 2013; Weissman-Fogel and Granovsky, 2019).
(IV) Non-invasive brain stimulation: In addition to TMS, transcranial direct current stimulation (tDCS) is also a common non-invasive brain stimulation technique for pain treatment (O’Connell et al., 2018; Lloyd et al., 2020; Pacheco-Barrios et al., 2020). tDCS is a non-invasive technology that uses a weak current (1−2 mA) to regulate the activity of neurons in the cerebral cortex. Existing studies have proved that both TMS and tDCS can effectively treat pain caused by different diseases (O’Connell et al., 2018). However, the comparative study of the two technologies is still lacking. Further research is needed to prove the difference and connection between the two technologies in the field of pain.

### Generally Accepted Conclusion Regarding Transcranial Magnetic Stimulation in Pain Research

(1) Definite analgesic effect of high-frequency rTMS of M1 contralateral to pain side in neuropathic pain (Level A). Low-frequency rTMS of M1 to pain side is probably ineffective in neuropathic pain (Level B). Possible analgesic effect of high-frequency rTMS of M1 contralateral to pain in complex regional pain syndrome type I (level C) (Lefaucheur et al., 2014). (2) There are inconclusive recommendations regarding rTMS of the dorsolateral prefrontal cortex (DLPFC) in fibromyalgia and neuropathic pain (Cruccu et al., 2016). (3) There is low-quality evidence that single doses of high-frequency rTMS of the motor cortex may have short-term effects on chronic pain (O’Connell et al., 2014).

### Future Research Trends

At present, TMS is still in the development stage of pain treatment, and future research can be carried out from the following aspects. First, it is necessary to explore the influencing factors of TMS in the treatment of pain. Second, we need to explore the mechanism of TMS in treating pain. Third, it is necessary to explore the clinical therapeutic effects of potential therapeutic targets.

### Limitations of This Study

To the best of our knowledge, this study is the first to access the trends of TMS in pain research based on literature published from 2010 to 2021 through a bibliometric approach. Nevertheless, this work has some limitations. Because of a limitation of the CiteSpace software, we only analyzed references in the WOS database. Some papers could inevitably have been missed. In addition, large-sample randomized controlled data are lacking.

## Conclusion

This study may help investigators discover the publication patterns and emerging trends of TMS on pain research from 2010 to 2021. The most influential author, institutions, journals, and countries were Jeanpascal Lefaucheur, University of São Paulo, *Clinical Neurophysiology*, and the United States. The visual map shows the hot research directions of TMS on pain research in recent years, such as TMS on neuropathic pain, motor cortex, and connectivity. Our bibliometrics analysis of 420 studies using CiteSpace software is in line with current clinical studies of TMS on pain research, indicating that the methodology is valid. In the future, large sample, randomized controlled trials are needed to carry out for TMS in the pain area.

## Data Availability Statement

The raw data supporting the conclusions of this article will be made available by the authors, without undue reservation.

## Author Contributions

ST contributed to the conception of the study. CL and MS performed the data analyses and wrote the manuscript. All authors contributed to the article and approved the submitted version.

## Conflict of Interest

The authors declare that the research was conducted in the absence of any commercial or financial relationships that could be construed as a potential conflict of interest.

## Publisher’s Note

All claims expressed in this article are solely those of the authors and do not necessarily represent those of their affiliated organizations, or those of the publisher, the editors and the reviewers. Any product that may be evaluated in this article, or claim that may be made by its manufacturer, is not guaranteed or endorsed by the publisher.
